# Early bolus epinephrine administration during pediatric cardiopulmonary resuscitation for bradycardia with poor perfusion: an ICU-resuscitation study

**DOI:** 10.1186/s13054-024-05018-7

**Published:** 2024-07-16

**Authors:** Amanda J. O’Halloran, Ron W. Reeder, Robert A. Berg, Tageldin Ahmed, Michael J. Bell, Robert Bishop, Matthew Bochkoris, Candice Burns, Joseph A. Carcillo, Todd C. Carpenter, J. Michael Dean, J. Wesley Diddle, Myke Federman, Richard Fernandez, Ericka L. Fink, Deborah Franzon, Aisha H. Frazier, Stuart H. Friess, Kathryn Graham, Mark Hall, David A. Hehir, Christopher M. Horvat, Leanna L. Huard, Martha F. Kienzle, Todd J. Kilbaugh, Tensing Maa, Arushi Manga, Patrick S. McQuillen, Kathleen L. Meert, Peter M. Mourani, Vinay M. Nadkarni, Maryam Y. Naim, Daniel Notterman, Murray M. Pollack, Anil Sapru, Carleen Schneiter, Matthew P. Sharron, Neeraj Srivastava, Bradley Tilford, Alexis A. Topjian, Shirley Viteri, David Wessel, Heather A. Wolfe, Andrew R. Yates, Athena F. Zuppa, Robert M. Sutton, Ryan W. Morgan

**Affiliations:** 1 Department of Anesthesiology and Critical Care Medicine, The Children’s Hospital of Philadelphia, University of Pennsylvania, 3401 Civic Center Boulevard, Philadelphia, PA 19104 USA; 2 Department of Pediatrics, University of Utah, Salt Lake City, UT USA; 3 Department of Pediatrics, Children’s Hospital of Michigan, Central Michigan University, Detroit, MI USA; 4 Department of Pediatrics, Children’s National Hospital, George Washington University School of Medicine, Washington, D.C., DC USA; 5 Department of Pediatrics, University of Colorado School of Medicine and Children’s Hospital Colorado, Aurora, CO USA; 6 Department of Critical Care Medicine, UPMC Children’s Hospital of Pittsburgh, University of Pittsburgh, Pittsburgh, PA USA; 7 Department of Pediatrics and Human Development, Michigan State University, Grand Rapids, MI USA; 8 Department of Pediatrics, Mattel Children’s Hospital, University of California Los Angeles, Los Angeles, CA USA; 9 Department of Pediatrics, Nationwide Children’s Hospital, The Ohio State University, Columbus, OH USA; 10 Department of Pediatrics, Benioff Children’s Hospital, University of California, San Francisco, San Francisco, CA USA; 11 Nemours Cardiac Center, Nemours Children’s Health, Wilmington, DE USA; 12 Department of Pediatrics, Sidney Kimmel Medical College, Thomas Jefferson University, Philadelphia, PA USA; 13 Department of Pediatrics, Washington University School of Medicine, St. Louis, MO USA; 14 Department of Pediatrics, University of Arkansas for Medical Sciences and Arkansas Children’s Hospital, Little Rock, AR USA; 15 Department of Molecular Biology, Princeton University, Princeton, NJ USA; 16 Department of Pediatrics, Nemours/Alfred I. duPont Hospital for Children and Thomas Jefferson University, Wilmington, DE USA

**Keywords:** Heart arrest, Cardiopulmonary resuscitation, Epinephrine, Bradycardia, Pediatric intensive care units, Hemodynamics

## Abstract

**Background:**

Half of pediatric in-hospital cardiopulmonary resuscitation (CPR) events have an initial rhythm of non-pulseless bradycardia with poor perfusion. Our study objectives were to leverage granular data from the ICU-RESUScitation (*ICU-RESUS*) trial to: (1) determine the association of early epinephrine administration with survival outcomes in children receiving CPR for bradycardia with poor perfusion; and (2) describe the incidence and time course of the development of pulselessness.

**Methods:**

Prespecified secondary analysis of *ICU-RESUS*, a multicenter cluster randomized trial of children (< 19 years) receiving CPR in 18 intensive care units in the United States. Index events (October 2016–March 2021) lasting ≥ 2 min with a documented initial rhythm of bradycardia with poor perfusion were included. Associations between early epinephrine (first 2 min of CPR) and outcomes were evaluated with Poisson multivariable regression controlling for a priori pre-arrest characteristics. Among patients with arterial lines, intra-arrest blood pressure waveforms were reviewed to determine presence of a pulse during CPR interruptions. The temporal nature of progression to pulselessness was described and outcomes were compared between patients according to subsequent pulselessness status.

**Results:**

Of 452 eligible subjects, 322 (71%) received early epinephrine. The early epinephrine group had higher pre-arrest severity of illness and vasoactive-inotrope scores. Early epinephrine was not associated with survival to discharge (aRR 0.97, 95%CI 0.82, 1.14) or survival with favorable neurologic outcome (aRR 0.99, 95%CI 0.82, 1.18). Among 186 patients with invasive blood pressure waveforms, 118 (63%) had at least 1 period of pulselessness during the first 10 min of CPR; 86 (46%) by 2 min and 100 (54%) by 3 min. Sustained return of spontaneous circulation was highest after bradycardia with poor perfusion (84%) compared to bradycardia with poor perfusion progressing to pulselessness (43%) and bradycardia with poor perfusion progressing to pulselessness followed by return to bradycardia with poor perfusion (62%) (*p* < 0.001).

**Conclusions:**

In this cohort of pediatric CPR events with an initial rhythm of bradycardia with poor perfusion, we failed to identify an association between early bolus epinephrine and outcomes when controlling for illness severity. Most children receiving CPR for bradycardia with poor perfusion developed subsequent pulselessness, 46% within 2 min of CPR onset.

**Supplementary Information:**

The online version contains supplementary material available at 10.1186/s13054-024-05018-7.

## Introduction

Pediatric in-hospital cardiac arrest (IHCA) is often the result of hemodynamic compromise secondary to progressive respiratory failure or shock accompanied by the development of bradycardia with poor perfusion [1, 2]. Expert consensus pediatric cardiac arrest guidelines recommend initiating cardiopulmonary resuscitation (CPR) for children with persistent non-pulseless bradycardia with poor perfusion as this may be a harbinger for pulseless cardiac arrest [3, 4]. Among children with IHCA, bradycardia with poor perfusion is the initial rhythm for which CPR is provided in approximately half of all events [5–7]. Similar to guidelines for pulseless cardiac arrest, epinephrine administration is recommended during pediatric CPR for bradycardia with poor perfusion [3, 4]. However, a propensity score matched analysis of pediatric IHCA registry data identified an association between epinephrine and worse survival outcomes in patients receiving CPR for an initial rhythm of bradycardia with poor perfusion, thus bringing this practice into question [8]. Though guidelines have not been changed to reflect this study’s results, they provide rationale for further study of this patient population.

Both physiologic premise and previously published data support timely epinephrine administration during pulseless cardiac arrest [9]. However, the utility of epinephrine in the subset of children receiving CPR for bradycardia with poor perfusion is unclear. Heterogeneity in the characteristics and outcomes of this population may confound the evaluation. First, epinephrine may preferentially be administered to children with a higher severity of illness and thus a lower likelihood of a favorable outcome, with limitations in the ability of registry data to account for these confounders. Second, many children with an initial rhythm of bradycardia with poor perfusion subsequently become pulseless [5–7].

While previous studies have included general data regarding the frequency with which clinicians detect subsequent pulselessness in children with an initial rhythm of bradycardia with poor perfusion [6, 7], data regarding the timing of pulselessness and evolution of cardiac rhythms throughout a CPR event are limited. Rhythm documentation in previous work is based on clinician documentation and retrospective data abstraction, potentially limiting accuracy. Moreover, the poor reliability of clinician pulse detection during cardiac arrest has also been described [10, 11]. In this patient population that has been reported to have different demographics, response to therapy (including epinephrine), and event outcomes compared to children with initial pulseless rhythms, there is utility in a more granular and data-driven description of cardiac rhythm progression and its association with outcomes using a less subjective method of rhythm determination.

The ICU-Resuscitation Project (*ICU-RESUS*) trial, a multicenter cluster randomized trial evaluating the effectiveness of physiology-focused CPR training on patient outcomes, collected Utstein-style data and physiologic waveforms for all enrolled patients [12, 13]. Our objective was to leverage this prospectively collected data to better understand pediatric cardiac arrests with an initial rhythm of bradycardia with poor perfusion. We first aimed to investigate the association between early epinephrine and outcomes in children receiving CPR for bradycardia with poor perfusion in the intensive care unit (ICU) setting. In the subset of patients with intra-arrest physiologic waveform data available, we aimed to also evaluate the incidence and time course of the development of pulselessness and determine the association between subsequent pulselessness and outcomes. We hypothesized that early epinephrine administration during CPR would be associated with higher rates of survival to hospital discharge with a favorable neurologic outcome when controlling for illness severity. Additionally, we hypothesized that most children receiving at least 2 min of CPR for an initial rhythm of bradycardia with poor perfusion would deteriorate to pulseless cardiac arrest.

## Methods

### Data source

This study is a pre-specified secondary analysis of *ICU-RESUS*, a parallel, hybrid, stepped-wedge, cluster randomized trial (ClinicalTrials.gov Identifier: NCT02837497) conducted in 18 United States (US) pediatric ICUs and pediatric cardiac ICUs across 10 clinical sites from October 1, 2016 to March 31, 2021. The *ICU-RESUS* trial analyzed the effectiveness of a physiology-focused cardiac arrest quality improvement bundle, including point-of-care training and cardiac arrest debriefing, on improving rates of survival to hospital discharge with a favorable neurologic outcome among pediatric IHCA patients. The methods and primary results have been previously published [12, 13]. The institutional review board at the University of Utah (Data Coordinating Center) and each clinical site approved the study with waiver of informed consent. We followed the Strengthening the Reporting of Observational Studies in Epidemiology (STROBE) reporting guideline (Supplemental Table 1) when writing our report [14].

### Study population

*ICU-RESUS* included index pediatric IHCA events occurring in children ≥ 37 weeks’ corrected gestational age and < 19 years of age who received chest compressions of any duration. Events were excluded if, prior to the arrest: (1) goals of care limited aggressive ICU therapies; (2) the patient was brain dead; or (3) the patient had an out-of-hospital cardiac arrest associated with the current hospitalization. For this secondary study, all included cases received chest compressions lasting ≥ 2 min and had an initial rhythm of bradycardia with poor perfusion documented by the clinical team. Two min was selected to exclude arrests with durations briefer than the window for our primary exposure. Patients were not required to receive epinephrine to be included. Patients receiving extracorporeal membrane oxygenation at the start of CPR were excluded. For inclusion in the subgroup analysis for the incidence and time course of the development of pulselessness based on invasive blood pressure (BP) waveform data, patients were required to have evaluable intra-arrest arterial BP waveforms.

### Data collection and study variables

Utstein-style data were collected at each study site by trained research coordinators, including patient demographics, pre-arrest characteristics, and event characteristics [15]. Baseline Pediatric Cerebral Performance Category (PCPC) scores and Functional Status Scale (FSS) scores were determined based on the subject’s status prior to the event leading to the current hospitalization [16, 17]. For subjects born during the current hospitalization or that had been hospitalized longer than 90 days at the time of the arrest, baseline PCPC and FSS were assessed based on the subject’s status prior to the decompensation associated with the cardiac arrest. Determination of initial CPR rhythm was based on the clinical team’s documentation. The vasoactive-inotropic score (VIS) was calculated 2 h prior to cardiac arrest [18, 19]. The Pediatric Risk of Mortality (PRISM) III score was determined 2–6 h prior to cardiac arrest [17]. For children with invasive arterial catheters in place, physiologic waveforms were collected for up to the first 10 min of CPR, de-identified, and transmitted to investigators at the Children’s Hospital of Philadelphia [20].

### Association between early epinephrine and outcomes

The primary exposure was “early” bolus-dose epinephrine, which was defined as epinephrine administration during the first 2 min of CPR. Epinephrine given within the first 2 min of CPR was selected a priori because the American Heart Association’s Get With The Guidelines—Resuscitation (GWTG-R) registry data indicate: (1) median time to epinephrine during pediatric IHCA is 1–2 min; and (2) children who develop subsequent pulselessness during CPR for an initial rhythm of bradycardia with poor perfusion do so at a median of 3 min [6, 8, 9]. By choosing epinephrine administration within 2 min as the primary exposure, we aimed to limit the number of events in the cohort with subjects who were already pulseless at the time they received epinephrine. The group that did not receive early bolus-dose epinephrine included events during which: (1) no epinephrine was administered or (2) the first dose of epinephrine was administered > 2 min after the start of CPR. The primary outcome was survival to hospital discharge with a favorable neurologic outcome, which was defined as a PCPC score of 1 (normal), 2 (mild disability), 3 (moderate disability), or no change from baseline. Exploratory outcomes included survival to hospital discharge, an alternative definition of survival to hospital discharge with a favorable neurologic outcome (defined as a PCPC score of 1, 2, or no change from baseline), and, among survivors, FSS score, PCPC at discharge, new morbidity (defined as an increase in FSS of 3 or greater from baseline to hospital discharge), and change from baseline FSS.

Patient and event characteristics were summarized according to whether early epinephrine was given. Frequencies and percentages were reported for categorical variables and medians and quartiles for continuous variables. Associations of summarized variables with early epinephrine were examined using Fisher’s exact test for categorical variables and the Wilcoxon rank-sum test for continuous variables. Patient and event characteristics and outcomes were also summarized based on CPR duration, comparing those events excluded due to CPR duration < 2 min to those with a CPR duration ≥ 2 min.

The associations between early epinephrine and outcomes were further investigated with Poisson regression with robust error estimates for binary outcomes and with ordinary linear regression for continuous outcomes. Covariates were selected a priori based on hypothesized or previously demonstrated associations with both administration of early epinephrine and the primary outcome. These included illness category (medical cardiac, medical non-cardiac, surgical cardiac, surgical non-cardiac, trauma), PRISM III score, VIS 2 h prior to CPR, presence of an epinephrine infusion at the start of CPR, hypotension as an immediate cause of the arrest, and respiratory decompensation as an immediate cause of the arrest [21–23]. Each of these covariates were individually included in the Poisson and linear regression models. Subgroup analyses using the same co-variates were conducted for: (1) patients with hypotension (and not respiratory decompensation) as an immediate cause of their arrest; (2) patients with respiratory decompensation (and not hypotension) as an immediate cause of their arrest; (3) patients with a cardiac illness category; and (4) neonates, as we hypothesized that epinephrine administration practices and response may differ in these subgroups.

### Invasive blood pressure waveform analyses

Investigators at the Children’s Hospital of Philadelphia (KG, RMS, RWM) reviewed arterial BP waveforms from the first 10 min of CPR as previously described [12, 20]. For the present study, the CPR rhythm during each interruption in chest compressions was evaluated and classified. Among the sub-group with invasive BP monitoring, CPR rhythm during interruptions was defined as ‘pulseless’ when there was either no deflection in the arterial waveform indicating a native beat or there was a native beat with a systolic BP (SBP) below the threshold at which a clinician would be expected to feel a pulse (i.e., pulseless electrical activity). These thresholds were set at SBP < 40 mmHg for infants (< 1 year of age) and < 50 mmHg for children ≥ 1 year of age [7, 11, 12, 20]. The CPR rhythm was classified as “non-pulseless” (i.e., bradycardia with poor perfusion) if during the interruption there was an arterial waveform deflection with SBP above the threshold for pulselessness. Due to the brevity of some interruptions, heart rate was unable to be calculated and therefore was not considered in characterizing CPR rhythms during chest compression interruptions. Each 30-s CPR epoch was classified as: (1) ongoing CPR for bradycardia with poor perfusion; (2) ongoing CPR for a pulseless rhythm; (3) ROSC; (4) transition to extracorporeal support; or (5) death. A stacked band plot was generated to visualize the CPR rhythms/outcomes of the cohort over the first 10 min of CPR. A sensitivity analysis was performed with events divided into those that received early epinephrine and those that did not.

### Association between subsequent pulselessness and outcomes

The primary exposure for this subgroup analysis of patients with invasive BP monitoring was the development of subsequent pulselessness during CPR initially provided for bradycardia with poor perfusion. Events were divided into three groups: (1) those with an initial rhythm of bradycardia with poor perfusion who did not develop pulselessness during the first 10 min of the CPR event; (2) those who did develop pulselessness; and (3) those who developed pulselessness and subsequently had at least one return to bradycardia with poor perfusion. The primary outcome for this analysis was sustained ROSC. Exploratory outcomes included survival to hospital discharge and survival to hospital discharge with a favorable neurologic outcome. Associations were measured using Fisher’s exact test for categorical variables and Kruskal–Wallis test for continuous variables. A sensitivity analysis was performed with events categorized by early epinephrine status.

All analyses were performed using SAS 9.4 (SAS Institute; Cary, NC). Reported p-values were based on a two-sided alternative and considered significant if less than 0.05.

## Results

**Fig. 1:**
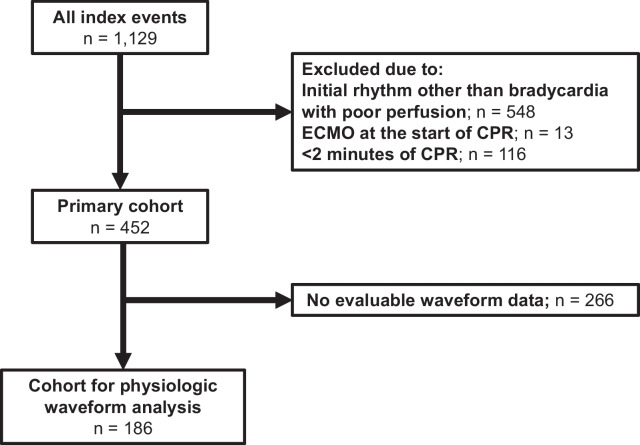
Study flow diagram

Of 1,129 index CPR events in *ICU-RESUS*, 452 met inclusion criteria and were included in the primary cohort (Fig. 1) with 66% (298/452) < 1 year of age. The majority of patients (62%; 280/452) had a primary cardiac illness category. Sixty-eight percent of patients had sustained ROSC. Fifty-seven percent of patients survived to hospital discharge, and 93% of survivors had a favorable neurologic outcome. Events excluded due to CPR duration < 2 min (n = 116) are described in Supplemental Tables 2 and 3. Compared to events with CPR duration ≥ 2 min, shorter events had lower median PRISM III scores (2 [IQR 0, 7) vs. 3 [IQR 0, 10], *p* = 0.001) and were more likely to be caused by respiratory decompensations (70% vs. 59%; *p* = 0.033). They were less likely to receive epinephrine (30% vs. 90%; *p* < 0.001) and had a higher rate of survival with a favorable neurologic outcome (75% vs. 52%; *p* < 0.01).

### Association between early epinephrine and outcomes

Table 1 compares demographics and characteristics in patients who received early epinephrine and those who did not. Seventy-one percent received early epinephrine (322/452). Age and illness category did not differ between the groups. Patients who received early epinephrine more frequently had pre-event hypotension (*p* < 0.001), sepsis (*p* = 0.01), and higher pre-arrest PRISM (*p* < 0.001) and VIS scores (*p* < 0.001). Supplemental Table 4 summarizes arterial BP during the first 2 min of CPR among patients with evaluable arterial BP data. Average systolic and diastolic BPs did not differ between the groups (*p* = 0.91 and *p* = 0.74, respectively).

**Table 1:** Demographics and patient characteristics

	Overall (N = 452)	Epinephrine bolus ≤ 2 min		*P*-value
		Yes (N = 322)	No (N = 130)	
*Demographics*				
Age				0.74
< 1 month	80 (18%)	60 (19%)	20 (15%)	
1 month—< 1 year	218 (42%)	150 (47%)	68 (52%)	
1 year—< 12 years	124 (27%)	90 (28%)	34 (26%)	
> 12 years	30 (7%)	22 (7%)	8 (6%)	
Weight (kg)	5.9 [3.6,9.8]	5.8 [3.7,12.5]	5.9 [3.6,9.8]	0.67
Male	242 (54%)	172 (53%)	70 (54%)	1.00
Race				0.65
White	195 (43%)	139 (43%)	56 (43%)	
Black or African American	118 (26%)	79 (25%)	39 (30%)	
Other	30 (7%)	20 (6%)	10 (8%)	
Unknown or Not Reported	109 (24%)	84 (26%)	25 (19%)	
Hispanic or Latino	67 (15%)	49 (15%)	18 (14%)	0.66
*Preexisting medical conditions*				
Respiratory insufficiency	406 (90%)	288 (89%)	118 (91%)	0.73
Congenital heart disease	289 (64%)	207 (64%)	82 (63%)	0.83
Congestive heart failure	60 (13%)	35 (11%)	25 (19%)	0.02
Pulmonary hypertension	84 (19%)	60 (19%)	24 (19%)	1.00
Pneumonia	53 (12%)	35 (11%)	18 (14%)	0.42
Sepsis	70 (16%)	59 (18%)	11 (9%)	0.01
Renal insufficiency	56 (12%)	43 (13%)	13 (10%)	0.43
Malignancy	17 (4%)	14 (4%)	3 (2%)	0.42
Trauma	9 (2%)	7 (2%)	2 (2%)	1.00
*Pre-event characteristics*				
Illness category				0.71
Medical cardiac	101 (22%)	67 (21%)	34 (26%)	
Medical non-cardiac	143 (32%)	104 (32%)	39 (30%)	
Surgical cardiac	179 (40%)	130 (40%)	49 (38%)	
Surgical non-cardiac	21 (5%)	16 (5%)	5 (4%)	
Trauma	8 (2%)	5 (2%)	3 (2%)	
PRISM	3.0 [0.0,10.0]	5.0 [0.0,11.0]	3.0 [0.0,7.0]	< 0.001
Vasoactive inotropic score	0.0 [0.0,7.0]	2.0 [0.0,8.0]	0.0 [0.0,4.0]	< 0.001
Baseline PCPC score				0.26
1	273 (60%)	187 (58%)	86 (66%)	
2	85 (19%)	69 (21%)	16 (12%)	
3	44 (10%)	31 (10%)	13 (10%)	
4	47 (10%)	33 (10%)	14 (11%)	
5	3 (1%)	2 (1%)	1 (1%)	
Baseline FSS	7.0 [6.0,10.0]	6.5 [6.0,10.0]	7.0 [6.0,11.0]	0.96
Interventions/devices in place prior to event				
Central venous catheter	310 (69%)	229 (71%)	81 (62%)	0.07
Invasive mechanical ventilation	325 (72%)	238 (74%)	87 (67%)	0.17
End-tidal CO_2_ monitoring	294 (65%)	217 (67%)	77 (59%)	0.10
Vasoactive infusion	245 (54%)	196 (61%)	49 (38%)	< 0.001
Epinephrine infusion 2 h prior to event	110 (24%)	91 (28%)	19 (15%)	0.002
Arterial catheter	236 (52%)	179 (56%)	57 (44%)	0.029
Non-invasive ventilation	84 (19%)	54 (17%)	30 (23%)	0.14

PRISM, Pediatric risk of mortality; PCPC,  Pediatric Cerebral Performance Category; FSS, Functional Status Scale; CO_2_, carbon dioxide.

Cardiac arrest event characteristics are summarized in Table 2. Ninety percent of the cohort (406/452) received at least one bolus dose of epinephrine during CPR. Among those who did not receive early epinephrine, 65% (84/130) received at least one bolus dose of epinephrine later during CPR. There were no differences between groups in the total number of doses of epinephrine (3 [IQR 1, 6] doses in the early epinephrine group vs 3 [IQR 1, 5.5] doses in the group that did not receive early epinephrine; *p* = 0.80) or the average interval between doses (4 [IQR 3.2, 5.8] min in the early epinephrine group vs. 4.6 min [IQR 3.1, 6.3] in the group that did not receive early epinephrine; *p* = 0.42). Those who received early epinephrine more frequently received calcium (48% vs. 34%; *p* = 0.01) and sodium bicarbonate (58% vs. 38%; *p* < 0.001). Fifteen percent of patients in each group received atropine (*p* = 1.00). The duration of CPR did not differ between groups: 9 [4,23] min in the early epinephrine group vs. 7.5 [3, 26] min in the no early epinephrine group (*p* = 0.88).

**Table 2:** Event characteristics

	Overall (N = 452)	Epinephrine bolus ≤ 2 min		P-value
		Yes (N = 322)	No (N = 130)	
*Immediate cause(s) of event*				
Respiratory decompensation	266 (59%)	182 (57%)	84 (65%)	0.14
Hypotension as immediate cause of event	253 (56%)	194 (60%)	59 (45%)	0.01
Arrhythmia	43 (10%)	30 (9%)	13 (10%)	0.86
Cyanosis without respiratory decompensation	21 (5%)	11 (3%)	10 (8%)	0.08
Duration of CPR (min)	9.0 [4.0,24.0]	9.0 [4.0,23.0]	7.5 [3.0,26.0]	0.88
*Category of Duration of CPR *(min)				0.74
< 6	175 (39%)	123 (38%)	52 (40%)	
6–15	113 (25%)	82 (26%)	31 (24%)	
16–35	77 (17%)	58 (18%)	19 (15%)	
> 35	87 (19%)	59 (18%)	28 (22%)	
*CPR time*^a^				0.53
Weekday	235 (52%)	162 (50%)	73 (56%)	
Weeknight	83 (18%)	62 (19%)	21 (16%)	
Weekend	134 (30%)	97 (30%)	36 (28%)	
*Pharmacologic interventions during event*				
Epinephrine	406 (90%)	322 (100%)	84 (65%)	< 0.001
Minutes to first dose^b^	1.0 [0.0.,2.0]	1.0 [0.0,1.0]	3.0 [3.0,6.0]	
Number of doses^b^	3.0 [1.0,6.0]	3.0 [1.0,6.0]	3.0 [1.0,5.5]	0.80
Average interval between doses^c^	4.1 [3.2,6.0]	4.0 [3.2,5.8]	4.6 [3.1,6.3]	0.42
Atropine	69 (15%)	49 (15%)	20 (15%)	1.00
Calcium	198 (44%)	154 (48%)	44 (34%)	0.01
Sodium bicarbonate	237 (52%)	187 (58%)	50 (39%)	< 0.001
Vasopressin	20 (4%)	13 (4%)	7 (5%)	0.61
Amiodarone	12 (3%)	7 (2%)	5 (4%)	0.34
Lidocaine	11 (2%)	7 (2%)	4 (3%)	0.52
Fluid bolus	124 (27%)	96 (30%)	28 (22%)	0.08

^a^Weekday is between 7 A.M. and 11 P.M. Monday–Friday; weeknight is after 11 P.M. Monday Thursday; weekend is from 11 P.M. on Friday through 7 A.M. on the following Monday

^b^Minutes to first dose of epinephrine and number of doses of epinephrine is only calculated on subjects who received at least 1 dose of epinephrine

^c^Average interval between epinephrine doses is only calculated on subjects with at least 2 doses of epinephrine

CPR, cardiopulmonary resuscitation.

Survival and exploratory functional outcomes did not differ between the groups in bivariate analyses (Supplemental Table 5). After adjusting for confounders, neither the primary outcome of survival to hospital discharge with a favorable neurologic outcome (RR 0.99 [95% CI 0.82, 1.18]; *p* = 0.89) nor any of the secondary outcomes were associated with early epinephrine administration (Table 3). Early epinephrine administration was not associated with survival with a favorable neurologic discharge among any of the analyzed subgroups (hypotension as immediate cause of arrest: aRR 1.03 [95% CI 0.67, 1.58]; *p* = 0.91; respiratory decompensation as immediate cause of arrest: aRR 0.85 [95% CI 0.68, 1.07]; *p* = 0.18; cardiac illness category: aRR 1.09 [95% CI 0.85, 1.39]; *p* = 0.52; neonates: aRR 1.03 [95% CI 0.68, 1.56]; *p* = 0.88) (Supplemental Tables 6–9).

**Table 3:** Association of early epinephrine bolus with outcomes

Outcome	Difference (95% CI)	Relative Risk (95% CI)	*P*-value
**Survival to hospital discharge with favorable neurologic outcome**^a^		**0.99 (0.82, 1.18)**	**0.89**
Sustained ROSC		0.98 (0.87, 1.12)	0.80
Survival to hospital discharge		0.97 (0.82, 1.14)	0.70
Survival to hospital discharge with PCPC of 1, 2, or no change from baseline		1.10 (0.89, 1.35)	0.39
Total FSS at hospital discharge	− 0.62 (− 1.82, 0.57)		0.31
PCPC at hospital discharge	− 0.02 (− 0.46, 0.41)		0.91
Change from baseline to hospital discharge in FSS of survivors	− 0.42 (− 1.32, 0.48)		0.36
New morbidity (survivors only)		0.78 (0.54, 1.12)	0.19

Bold value indicates the primary outcome

^a^Favorable neurologic outcome was defined as a PCPC of 1, 2, 3, or no change from baseline

FSS, Functional Status Scale; PCPC, Pediatric Cerebral Performance Category

### Incidence and timing of subsequent pulselessness

**Fig. 2:**
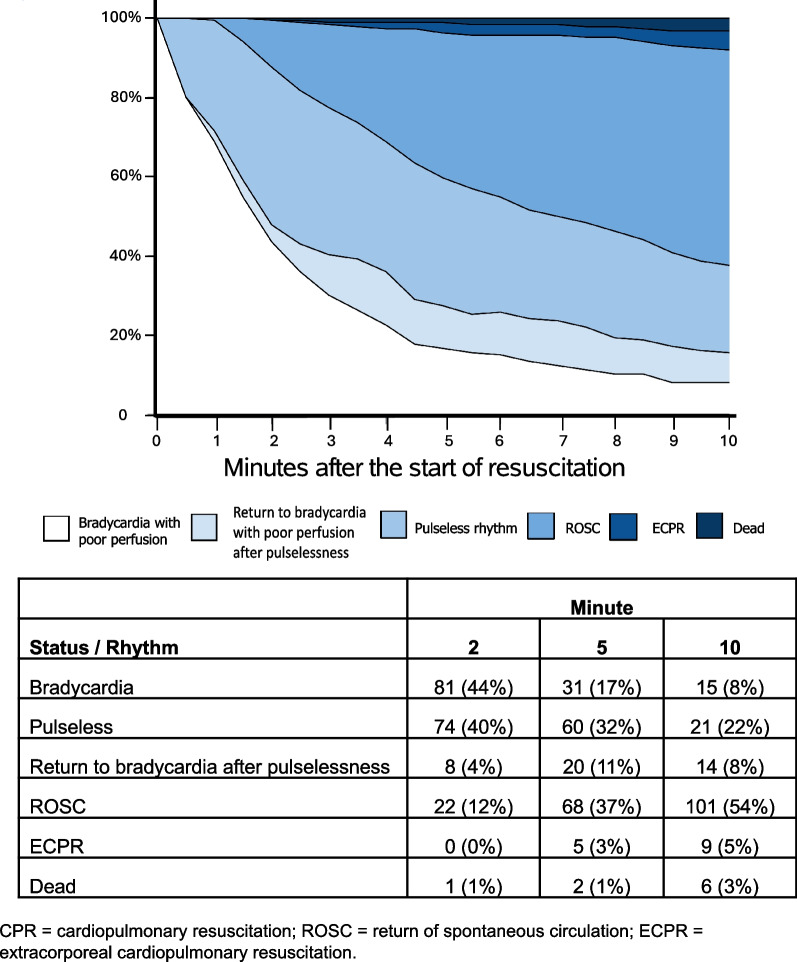
Temporal evolution of CPR rhythms and outcomes. Subjects include the 186 patients in the cohort with evaluable arterial line waveform data. Stacked band plot of subjects' rhythm status over the first 10 minutes of CPR. A cross-section at any moment in time shows the percentage of subjects iwth each status at that time

Among 186 events with invasive BP data, 118 (63%) had at least one period of pulselessness during the first 10 min of CPR; 57/186 (31%) patients experienced pulselessness by 1 min, 86/186 (46%) by 2 min, 100/186 (54%) by 3 min, and 113/186 (61%) by 5 min (Supplemental Table 10). Figure 2 depicts patient status at each minute based on analysis of chest compression interruptions during the first 10 min of CPR. Of the 186 events with invasive BP data, 179 (96%) received bolus-dose epinephrine. Twenty-nine percent (52/179) experienced at least one period of pulselessness in or prior to the minute that they received the first bolus dose of epinephrine. The temporal evolution of pulselessness was similar when dividing the cohort into those that received early epinephrine and those that did not (Supplemental Figs. 1 and 2).

### Association between subsequent pulselessness and outcomes

Among patients with invasive BP monitoring, those who never developed pulselessness had the highest rate of sustained ROSC on bivariate analysis (57/68; 84%; Table 4), followed by those who developed pulselessness and subsequently had at least one return to bradycardia with poor perfusion (33/53; 62%), and those who developed pulselessness without a subsequent return to bradycardia with poor perfusion (28/65; 43%) (*p* < 0.001). There were no significant differences in rates of survival to hospital discharge (*p* = 0.10) or survival to hospital discharge with a favorable neurologic outcome (*p* = 0.07) between these groups.

**Table 4:** Association of subsequent pulselessness status with outcomes

	Never developed pulselessness (n = 68)	Developed subsequent pulselessness (n = 65)	Developed pulselessness and subsequently had at least one return to bradycardia with poor perfusion (n = 53)	*P*-value
**Sustained ROSC**	**57 (84%)**	**28 (43%)**	**33 (62%)**	** < 0.001**
Survival to hospital discharge	45 (66%)	31 (48%)	30 (57%)	0.10
Survival to hospital discharge with favorable neurologic outcome^a^	45 (66%)	30 (46%)	29 (55%)	0.07
Survival to hospital discharge with PCPC of 1, 2, or no change from baseline	42 (62%)	26 (40%)	27 (51%)	0.045

Bold value indicates the primary outcome

Subsequent pulselessness status of the 186 patients in the cohort with evaluable arterial blood pressure data are included

^a^Favorable neurologic outcome was defined as a PCPC of 1, 2, 3, or no change from baseline

ROSC, return of spontaneous circulation

The sensitivity analysis with events divided based on early epinephrine status is summarized in Supplemental Table 11. Among events with early epinephrine administration, sustained ROSC remained associated with subsequent pulselessness status with the highest rates of ROSC (85%) among those patients who never developed pulselessness (*p* < 0.001). There was no significant association identified between survival to hospital discharge or survival with favorable neurologic outcome in either the early epinephrine or no early epinephrine groups.

## Discussion

In this multicenter study, we failed to identify an association between early bolus dose epinephrine administration and rates of survival to hospital discharge with a favorable neurologic outcome among children receiving CPR for an initial rhythm of bradycardia with poor perfusion. Among patients with available invasive BP waveform data, we established that nearly half (46%) experienced at least one period of pulselessness during the first 2 min of CPR after an initial rhythm of bradycardia with poor perfusion. Our analysis leveraging the robust *ICU-RESUS* dataset to study pediatric IHCA with an initial rhythm of bradycardia with poor perfusion supports: (1) that further investigation is needed to determine the effect of epinephrine in this patient population; and (2) that bradycardia with poor perfusion is often a harbinger of rapid deterioration to pulselessness.

Epinephrine’s α-adrenergic vasopressor effects increase coronary perfusion pressure, contributing to achieving ROSC during cardiac arrest. Moreover, epinephrine’s potent β-adrenergic properties can improve cardiac output by heart rate augmentation and intropy, potentially important mechanisms for children with ongoing but compromised native cardiac output. Despite this strong physiologic premise, Holmberg et al*.* found a surprising association between epinephrine and worse survival outcomes among children receiving CPR for bradycardia with poor perfusion in the GWTG-R registry [8]. We hypothesized that utilizing prospectively collected data and accounting for additional markers of severity of illness (PRISM III score, VIS, and presence of an epinephrine infusion at the start of CPR) would decrease confounding that may have contributed to this finding. Additionally, as these patients frequently progress to pulselessness in the first minutes of CPR, we used *early* epinephrine administration as our primary exposure in an effort to limit reverse causation bias (i.e., patients receiving epinephrine after 2 min were likely to already be pulseless at the time of administration). However, we failed to identify an association between early epinephrine and improved rates of survival with a favorable neurologic outcome. A possible explanation for these findings, which conflict with both our hypothesis and the previous study, lies in the heterogeneity of the population of children with an initial CPR rhythm of bradycardia with poor perfusion.

It is increasingly clear that the efficacy of epinephrine during cardiac arrest is not uniform. A separate, prospectively designed, secondary analysis of the *ICU-RESUS* trial data analyzed the association between physiologic responsiveness to epinephrine and survival outcomes. That study identified substantial heterogeneity in the change in invasively measured diastolic BP in response to epinephrine administration (median change of 4.4 mmHg [IQR − 1.9, 11.5]), with 45% of patients classified as epinephrine responders (defined as a DBP change of ≥ 5 mmHg) [20]. Thus, regardless of initial rhythm, some patients achieve the intended physiologic effects of epinephrine during CPR while others do not. As such, epinephrine is likely beneficial for some children with an initial rhythm of bradycardia with poor perfusion and not for others. Differences between our cohort (ICU patients at academic referral centers, many with pre-event invasive interventions in place, and a high proportion of congenital heart disease) and the GWTG-R cohort may partially explain our differing findings. We also conducted subgroup analyses of events based on immediate arrest etiology (hypotension and respiratory decompensation), since etiology is an additional factor not easily obtained from most registry data and we hypothesized that it would have an effect on the association between survival outcomes and early bolus epinephrine administration. While we did not find an association between early bolus epinephrine and survival to discharge in either subgroup, further investigation to better understand patient populations likely to benefit from specific cardiac arrest therapies is a potentially fruitful area for future study.

In this secondary analysis of a cluster randomized trial, we must also consider the potential impact of the trial intervention on the exposure or outcomes in question. The *ICU-RESUS* trial studied a resuscitation quality improvement bundle, which could be expected to result in variable resuscitation practices (including epinephrine administration) and outcomes (including survival to hospital discharge) [12]. However, achieving target chest compression rate, depth, and fraction (markers of CPR quality) did not differ between the parent trial intervention and control groups (rate: OR 1.3 [95% CI 0.65–2.63], *p* = 0.46; depth: OR 1.28 [95% CI 0.52–3.24], *p* = 0.59; fraction: OR 0.82 [95% CI 0.51–1.33], *p* = 0.42), nor did event outcomes (survival to discharge with favorable neurologic outcome: aOR 1.08 [95% CI 0.76–1.53; survival to discharge: aOR 1.03 [95% CI 0.73–1.47]). More specifically, when dividing our cohort by treatment allocation, there were no differences in the percentage of cases receiving epinephrine (control: 91%, treatment: 89%; *p* = 0.431), time to first epinephrine (control: 1 min [IQR 0, 2], treatment: 1 min [IQR 0, 2], or total number of doses (control: 3 [IQR 1, 5], treatment: 2 [IQR 1, 7]; *p* = 0.476). Considering the lack of differences noted between the groups in these relevant outcomes and metrics, we expect it is unlikely that intervention allocation confounded this study although unmeasured confounding remains a possibility.

To increase our understanding of children receiving CPR who have an initial rhythm of bradycardia with poor perfusion, we analyzed the *ICU-RESUS* BP waveforms to present a granular summary of rhythm status and pulselessness during the first 10 min of CPR. Similar to earlier studies reliant on clinician reporting of subsequent cardiac arrest rhythms, we confirmed that the majority of children with an initial rhythm of bradycardia with poor perfusion deteriorate to pulselessness. Additionally, this happens quickly, often by the time of a guideline-recommended first pulse check (54% of those with invasive BP monitoring experienced pulselessness within 3 min of starting CPR). Notably, nearly one-third of the patients who received any bolus doses of epinephrine experienced pulselessness before or during the minute in which they first received epinephrine. This suggests that many patients in previous work may have already been pulseless at the time of epinephrine administration, which may have biased toward identifying worse outcomes.

Our findings should be interpreted in light of several limitations. All sites included in the parent study are ICUs at academic referral centers in the US and the majority of patients (62%) had a cardiac illness category, both of which could impact the generalizability of our results. The study design is observational, preventing any determination of causality between the exposure and outcomes. There were an insufficient number of patients to perform time-dependent propensity score matching, which was the methodology utilized by the aforementioned GWTG-R study. There could be inaccuracies in documentation of the timing of epinephrine administration that would impact exposure group assignment, though the nesting of this study within the structure of a prospective trial likely improves data accuracy compared with most pediatric cardiac arrest studies. The exact bolus dose of epinephrine administered was not available, limiting our ability to consider the impact of doses that deviate from the guideline recommendations. The exposure of ‘early epinephrine’ (given within 2 min of the start of CPR) was selected to limit the number of patients in the cohort who had already deteriorated to pulselessness by the time of epinephrine administration and therefore would be at higher risk for a worse outcome regardless of epinephrine status. Inevitably, as confirmed in our subset of patients with invasive BP data, many patients had already deteriorated to pulselessness at 2 min (i.e., received epinephrine with a rhythm of PEA rather than bradycardia with poor perfusion). By excluding events with CPR duration < 2 min, we could be introducing bias by excluding a subgroup of patients whose rapid ROSC may be partially attributable to epinephrine. However, descriptive statistics of this subgroup show that compared to events with duration ≥ 2 min, they had lower PRISM III scores, were much less likely to receive epinephrine (30% vs. 90%), and had better patient outcomes, suggesting that they are instead less ‘severe’ events with a higher likelihood of ROSC compared to the events in our cohort regardless of epinephrine administration. Many of the patients in the study (72%) had an invasive airway in place prior to arrest; continuous chest compressions provided in 2-min intervals without intervening pulse checks (as recommended by cardiac arrest guidelines) may have biased our analysis toward identifying pulselessness later in these patients. Epinephrine administration was common but not universal among patients who did not receive ‘early epinephrine’ group (65%), and the median time for first dose was 3 min. While analyzing patients who received ‘later’ epinephrine and those who did not receive epinephrine as one group deserves consideration in interpretation of our results, our chosen exposure of whether a patient received early epinephrine without consideration for events occurring after the time point in question reflects clinician intra-arrest knowledge. Only a small percentage of events had invasive BP data available, resulting in a much smaller sample size for the secondary analyses of these events. In addition, all patients in the invasive BP analysis had arterial lines. While arterial lines are often in place among children with cardiac arrests in the ICU, the pre-arrest presence of this invasive monitoring modality suggests higher acuity in this subset of events [24].

## Conclusions

In this cohort of pediatric IHCA with an initial rhythm of bradycardia with poor perfusion, we did not identify an association between early bolus epinephrine and outcomes when controlling for illness severity, which differs from a previous investigation showing an association between epinephrine and worse outcomes in these patients [8]. Many children receiving CPR for bradycardia with poor perfusion progress to pulselessness and do so quickly, with forty-six percent of children becoming pulseless within 2 min of the start of CPR. These data support additional studies to further support or refute current guideline recommendations for the administration of epinephrine during CPR in this population.

### Supplementary Information

Supplementary Material.

## Data Availability

The dataset from the ICU Resuscitation trial is available for public use via the National Institutes of Health (NIH) Biologic Specimen and Data Repository Information Coordinating Center (BioLINCC).

## Abbreviations

BP: Blood pressure
CPR: Cardiopulmonary resuscitation
FSS: Functional Status Scale
GWTG-R: Get With The Guidelines—Resuscitation
IHCA: In-hospital cardiac arrest
ICU: Intensive care unit
PCPC: Pediatric Cerebral Performance Category
PRISM: Pediatric Risk of Mortality
SBP: Systolic blood pressure
US: United States
VIS: Vasoactive-inotropic score
