# Supplementary material for: Early bolus epinephrine administration during pediatric cardiopulmonary resuscitation for bradycardia with poor perfusion: an ICU-resuscitation study
Source: Crit Care. 2024 Jul 16;28:242. doi: 10.1186/s13054-024-05018-7 (PMC11251231; doi:10.1186/s13054-024-05018-7)
Supplement: Supplementary file 1 — Supplementary Material. [file 13054_2024_5018_MOESM1_ESM.docx]

**Supplemental Material**

**Supplemental Table 1.** Completed STROBE Checklist

**Supplemental Table 2.** Patient and Event Characteristics by CPR Duration, including patients with CPR duration < 2 minutes

**Supplemental Table 3.** Summary of Outcomes by CPR Duration, including patients with CPR duration < 2 minutes

**Supplemental Table 4.** Blood Pressure During First Two Minutes of CPR Among Patients with Evaluable Arterial Line Data

**Supplemental Table 5.** Univariate Association of Early Epinephrine Bolus with Outcomes

**Supplemental Table 6.** Outcomes in Patients with Hypotension as Immediate Cause of Arrest

**Supplemental Table 7.** Outcomes in Patients with Respiratory Decompensation as Immediate Cause of Arrest

**Supplemental Table 8.** Association of Early Epinephrine Bolus with Outcomes in Patients with Cardiac Illness Category

**Supplemental Table 9.** Association of Early Epinephrine Bolus with Outcomes in Neonates

**Supplemental Table 10.** Cumulative Percent of Subjects with Rhythm Status Prior to the Current Minute

**Supplemental Figure 1.** Temporal Evolution of CPR Rhythms and Outcomes, patients receiving early epinephrine

**Supplemental Figure 2.** Temporal Evolution of CPR Rhythms and Outcomes, patients not receiving early epinephrine

**Supplemental Table 11.** Association of Subsequent Pulselessness Status with Outcomes, patients categorized by early epinephrine status

**Supplemental Table 1.** Completed STROBE Checklist for Observational Studies

|  | Item No | Recommendation |
| --- | --- | --- |
| **Title and abstract** | 1 | (*a*) Indicate the study’s design with a commonly used term in the title or the abstract  Prespecified secondary analysis of ICU-RESUS as stated in the Abstract and the Methods. |
|  |  | (b) Provide in the abstract an informative and balanced summary of what was done and what was found  Provided in the Abstract. |
| Introduction | | |
| Background/rationale | 2 | Explain the scientific background and rationale for the investigation being reported  Included in the Introduction. |
| Objectives | 3 | State specific objectives, including any prespecified hypotheses  Included in the Introduction. |
| Methods | | |
| Study design | 4 | Present key elements of study design early in the paper  Included in the Methods. |
| Setting | 5 | Describe the setting, locations, and relevant dates, including periods of recruitment, exposure, follow-up, and data collection  Included in the Methods. |
| Participants | 6 | (a) Give the eligibility criteria, and the sources and methods of selection of participants. Describe methods of follow-up  Included in the Methods. |
|  |  | (b) For matched studies, give matching criteria and number of exposed and unexposed  Not applicable. |
| Variables | 7 | Clearly define all outcomes, exposures, predictors, potential confounders, and effect modifiers. Give diagnostic criteria, if applicable  Included in the Methods. |
| Data sources/ measurement | 8* | For each variable of interest, give sources of data and details of methods of assessment (measurement). Describe comparability of assessment methods if there is more than one group  Included in the Methods. |
| Bias | 9 | Describe any efforts to address potential sources of bias  Addressed in the Methods and in the Discussion. |
| Study size | 10 | Explain how the study size was arrived at  Included in the Methods. |
| Quantitative variables | 11 | Explain how quantitative variables were handled in the analyses. If applicable, describe which groupings were chosen and why  Included in the Methods. |
| Statistical methods | 12 | (a) Describe all statistical methods, including those used to control for confounding  Included in the Methods. |
|  |  | (b) Describe any methods used to examine subgroups and interactions  Included in the Methods. |
|  |  | (c) Explain how missing data were addressed  Included in the Methods and in Figure 1. |
|  |  | (d) If applicable, explain how loss to follow-up was addressed  Not applicable. |
|  |  | (e) Describe any sensitivity analyses  Included in the Methods. |
| Results | | |
| Participants | 13* | (a) Report numbers of individuals at each stage of study—eg numbers potentially eligible, examined for eligibility, confirmed eligible, included in the study, completing follow-up, and analysed  Included in Figure 1. |
|  |  | (b) Give reasons for non-participation at each stage  Included in Figure 1. |
|  |  | (c) Consider use of a flow diagram  Included as Figure 1. |
| Descriptive data | 14* | (a) Give characteristics of study participants (eg demographic, clinical, social) and information on exposures and potential confounders  Included in the Results and in Tables 1 and 2. |
|  |  | (b) Indicate number of participants with missing data for each variable of interest  Included in Figure 1. |
|  |  | (c) Summarise follow-up time (eg, average and total amount)  Not applicable. |
| Outcome data | 15* | Report numbers of outcome events or summary measures over time  Included in the Results and in Tables 1-2 and Supplemental Tables 3-5. |
| Main results | 16 | (a) Give unadjusted estimates and, if applicable, confounder-adjusted estimates and their precision (eg, 95% confidence interval). Make clear which confounders were adjusted for and why they were included  Included in the Methods, in the Results, and in Tables 3-4 and Supplemental Tables 4-5. |
|  |  | (b) Report category boundaries when continuous variables were categorized  Included in the Results and in Tables 1-2 and Supplemental Table 3. |
|  |  | (c) If relevant, consider translating estimates of relative risk into absolute risk for a meaningful time period  Not applicable. |
| Other analyses | 17 | Report other analyses done—eg analyses of subgroups and interactions, and sensitivity analyses  Included in the Results and in Supplemental Tables 4-5. |
| Discussion | | |
| Key results | 18 | Summarise key results with reference to study objectives  Included in the Discussion. |
| Limitations | 19 | Discuss limitations of the study, taking into account sources of potential bias or imprecision. Discuss both direction and magnitude of any potential bias  Included in the Discussion and Conclusions. |
| Interpretation | 20 | Give a cautious overall interpretation of results considering objectives, limitations, multiplicity of analyses, results from similar studies, and other relevant evidence  Included in the Discussion. |
| Generalisability | 21 | Discuss the generalisability (external validity) of the study results  Included in the Discussion. |
| Other information | | |
| Funding | 22 | Give the source of funding and the role of the funders for the present study and, if applicable, for the original study on which the present article is based  Included in the Declarations section. |

*Give information separately for exposed and unexposed groups.

**Supplemental Table 2.** Patient and Event Characteristics by CPR Duration, including patients with CPR duration <2 minutes

|  |  | **CPR Duration** | |  |
| --- | --- | --- | --- | --- |
|  | **Overall**  (N = 568) | **Duration <2 min**  (N = 116) | **Duration ≥2 min**  (N = 452) | **P-value** |
| **Demographics** |  |  |  |  |
| Age |  |  |  | 0.891 |
| <1 month | 100 (18% | 20 (17%) | 80 (18% |  |
| 1 month - <1 year | 278 (49%) | 60 (52%) | 218 (48%) |  |
| 1 year - <12 years | 152 (27%) | 28 (24%) | 124 (27%) |  |
| >12 years | 38 (7%) | 8 (7%) | 30 (7%) |  |
| Weight (kg) | 5.8 (3.8, 11.0) | 5.6 (4.0, 9.7) | 5/9 (3.7, | 0.836 |
| Male | 304 (54%) | 62 (53%) | 242 (54%) | 1.00 |
| Race |  |  |  | 0.376 |
| White | 250 (44%) | 55 (47%) | 195 (43%) |  |
| Black or African American | 153 (26%) | 35 (30%) | 118 (26%) |  |
| Other | 34 (6%) | 4 (3%) | 30 (7%) |  |
| Unknown or Not Reported | 131 (23%) | 22 (19%) | 109 (24%) |  |
| Hispanic or Latino | 85 (15%) | 18 (16%) | 67 (15%) | 0.884 |
| Preexisting medical conditions |  |  |  |  |
| Respiratory insufficiency | 506 (89%) | 100 (86%) | 406 (90%) | 0.316 |
| Congenital heart disease | 365 (64%) | 76 (66%) | 289 (64%) | 0.828 |
| Congestive heart failure | 67 (12%) | 7 (6%) | 60 (13% | 0.035 |
| Pulmonary hypertension | 105 (19%) | 21 (18%) | 84 (19%) | 1.00 |
| Pneumonia | 62 (11%) | 9 (8%) | 53 (12%) | 0.247 |
| Sepsis | 80 (14%) | 10 (9%) | 70 (16%) | 0.072 |
| Renal insufficiency | 65 (11%) | 9 (8%) | 56 (12%) | 0.192 |
| Malignancy | 19 (3.3%) | 2 (2%) | 17 (4%) | 0.390 |
| Trauma | 14 (3%) | 5 (4%) | 9 (2%) | 0.176 |
| **Pre-event characteristics** |  |  |  |  |
| Illness category |  |  |  | 0.378 |
| Medical cardiac | 129 (23%) | 29 (24%) | 101 (22%) |  |
| Medical non-cardiac | 183 (32%) | 40 (35%) | 143 (32%) |  |
| Surgical cardiac | 217 (38%) | 38 (33%) | 179 (40%) |  |
| Surgical non-cardiac | 26 (5%) | 5 (4%) | 21 (5%) |  |
| Trauma | 13 (2%) | 5 (4%) | 8 (2%) |  |
| PRISM | 3.0 (0, 9.0) | 2.0 (0. 7.0) | 3.0 (0, 10.0) | 0.001 |
| Vasoactive inotropic score | 0 (0, 6.0) | 0 (0, 2.5) | 0 (0, 7.0) | <0.001 |
| Baseline PCPC score |  |  |  | 0.567 |
| 1 | 347 (61%) | 74 (64%) | 273 (60%) |  |
| 2 | 103 (18%) | 18 (16%) | 85 (19%) |  |
| 3 | 57 (10%) | 13 (11%) | 44 (10% |  |
| 4 | 57 (10%) | 10 (9%) | 47 (10%) |  |
| 5 | 4 (0.7%) | 1 (0.9%) | 3 (0.7% |  |
| Baseline FSS | 6.0 (6.0, 10.0) | 6.0 (6.0, 10.5) | 7.0 (6.0, 10.0) | 0.438 |
| **Interventions/devices in place prior to event** |  |  |  |  |
| Central venous catheter | 384 (68%) | 74 (64%) | 310 (69%) | 0.374 |
| Invasive mechanical ventilation | 402 (71%) | 77 (66%) | 325 (72%) | 0.254 |
| End-tidal CO_2_ monitoring | 363 (64%) | 69 (60%) | 294 (65%) | 0.280 |
| Vasoactive infusion | 286 (50%) | 41 (35%) | 245 (54%) | <0.001 |
| Non-invasive ventilation | 104 (18%) | 20 (17%) | 84 (19%) | 0.789 |
| **Immediate cause(s) of event** |  |  |  |  |
| Respiratory decompensation | 347 (61%) | 81 (70%) | 266 (59%) | 0.033 |
| Hypotension as immediate cause of event | 289 (51%) | 36 (31%) | 253 (56%) | <0.001 |
| Arrhythmia | 54 (10%) | 11 (10%) | 43 (10%) | 1.00 |
| Cyanosis without respiratory decompensation | 26 (5%) | 5 (4%) | 21 (5%) | 1.00 |
| **Duration of CPR (minutes)** | 5 (2, 19) | 1 (1, 1) | 9 (4, 24) | <0.001 |
| **Category of Duration of CPR (minutes)** |  |  |  | <0.001 |
| <6 | 291 (51%) | 116 (100%) | 175 (39%) |  |
| 6-15 | 113 (20%) | 0 (0%) | 113 (25%) |  |
| 16-35 | 77 (14%) | 0 (0%) | 77 (17%) |  |
| >35 | 87 (15%) | 0 (0%) | 87 (19%) |  |
| **CPR time^a^** |  |  |  | 0.447 |
| Weekday | 298 (53%) | 63 (54%) | 235 (52%) |  |
| Weeknight | 108 (19%) | 25 (22%) | 83 (18%) |  |
| Weekend | 162 (29%) | 28 (24%) | 134 (30%) |  |
| **Pharmacologic interventions during event** |  |  |  |  |
| Epinephrine | 441 (78%) | 35 (30%) | 406 (90%) | <0.001 |
| Minutes to first dose^b^ | 1 (0, 2) | 0 (0, 0) | 1 (0, 2) |  |
| Number of doses^b^ | 2 (1, 5) | 1 (1, 1) | 3 (1, 6) | <0.001 |
| Average interval between doses^c^ | 4.1 (3.1, 6.0) | 1.0 (0, 1.0) | 4.1 (3.2, 6.0) | 0.004 |
| Atropine | 79 (14%) | 10 (9%) | 69 (15%) | 0.071 |
| Calcium | 203 (36%) | 5 (4%) | 198 (44%) | <0.001 |
| Sodium bicarbonate | 241 (42%) | 4 (3%) | 237 (52%) | <0.001 |
| Vasopressin | 21 (4%) | 1 (0.9%) | 20 (4%) | 0.095 |
| Amiodarone | 12 (2%) | 0 (0%) | 12 (3%) | 0.139 |
| Lidocaine | 11 (2%) | 0 (0%) | 11 (2%) | 0.132 |
| Fluid bolus | 129 (23%) | 5 (4%) | 124 (27%) | <0.001 |

kg = kilograms; PRISM = Pediatric Risk of Mortality Score; PCPC = Pediatric Cerebral Performance Category; FSS = Functional Status Scale; CO_2_ = carbon dioxide; CPR = cardiopulmonary resuscitation

^a^Weekday is between 7 AM and 11 PM Monday - Friday; weeknight is after 11 PM Monday Thursday; Weekend is from 11 PM on Friday through 7 AM on the following Monday.

^b^Minutes to first dose of epinephrine and number of doses of epinephrine is only calculated on subjects who received at least 1 dose of epinephrine.

^c^Average interval between epinephrine doses is only calculated on subjects with at least 2 doses of epinephrine.

CPR = cardiopulmonary resuscitation

**Supplemental Table 3.** Summary of Outcomes by CPR Duration, including patients with CPR duration < 2 minutes

|  | **CPR Duration** | |  |
| --- | --- | --- | --- |
|  | **Duration <2 min**  (N = 116) | **Duration ≥2 min**  (N = 452) | **P-value** |
| **Survival to hospital discharge with favorable neurologic outcome^a^** | 87 (75%) | 239 (52%) | <0.01 |
| Sustained ROSC | 115 (99%) | 307 (68%) | <0.01 |
| Survival to hospital discharge | 94 (81%) | 256 (57%) | <0.01 |
| Survival to hospital discharge with PCPC of 1, 2, or no worse from baseline | 81 (70%) | 217 (48%) | <0.01 |
| PCPC at hospital discharge |  |  | <0.01 |
| 1 | 37 (32%) | 110 (24%) |  |
| 2 | 29 (25%) | 70 (15%) |  |
| 3 | 14 (12%) | 39 (9%) |  |
| 4 | 14 (12%) | 34 (8%) |  |
| 5 | 0 (0%) | 3 (1% |  |
| Change from baseline to hospital discharge in FSS of survivors | 22 (19% | 1 (0, 3) | 0.74 |
| New morbidity (survivors only) | 28 (30%) | 82 (32%) | 0.80 |

^a^Favorable neurologic outcome was defined as a PCPC of 1, 2, 3, or no change from baseline.

**Supplemental Table 4.** Blood Pressure During First Two Minutes of CPR Among Patients with Evaluable Arterial Line Data

|  | **Epinephrine bolus ≤2 minutes** | | **P-value** |
| --- | --- | --- | --- |
|  | **Yes** | **No** |  |
| **Average DBP (mmHg; median [IQR])** | 35 [27, 45] | 35 [30, 41] | 0.91 |
| Age <1 year | 34 [26, 44] | 33 [27, 38] | 0.66 |
| Age ≥1 year | 39 [28, 46] | 41 [38, 49] | 0.27 |
| **Average SBP (mmHg; median [IQR])** | 76 [56, 96] | 73 [59, 92] | 0.74 |
| Age <1 year | 72 [56, 94] | 67 [58, 87] | 0.65 |
| Age ≥1 year | 84 [63, 105] | 84 [62, 112] | 0.70 |

CPR = cardiopulmonary resuscitation; DBP = diastolic blood pressure; IQR = interquartile range; SBP = systolic blood pressure

**Supplemental Table 5.** Univariate Association of Early Epinephrine Bolus with Outcomes

|  | **Epinephrine bolus ≤2 minutes** | | **P-value** |
| --- | --- | --- | --- |
|  | **Yes**  (N = 322) | **No**  (N = 130) |  |
| **Survival to hospital discharge with favorable neurologic outcome^a^** | **164 (51%)** | **75 (58%)** | **0.21** |
| Sustained ROSC | 213 (66%) | 94 (72%) | 0.22 |
| Survival to hospital discharge | 174 (54%) | 82 (63%) | 0.09 |
| Survival to hospital discharge with PCPC of 1, 2, or no change from baseline | 154 (48%) | 63 (48%) | 0.92 |
| PCPC at hospital discharge |  |  | 0.36 |
| 1 | 76 (24%) | 34 (26%) |  |
| 2 | 56 (17%) | 14 (11%) |  |
| 3 | 21 (7%) | 18 (14%) |  |
| 4 | 19 (6%) | 15 (12%) |  |
| 5 | 2 (1%) | 1 (1%) |  |
| 6 | 148 (46%) | 48 (37%) |  |
| Total FSS at hospital discharge | 9 [7, 12] | 10 [6, 14] | 0.16 |
| Change from baseline to hospital discharge in FSS of survivors | 1 [0, 3] | 1 [0, 5] | 0.92 |
| New morbidity (survivors only) | 52 (30%) | 30 (37%) | 0.32 |

^a^Favorable neurologic outcome was defined as PCPC of 1, 2, 3, or no change from baseline.

PCPC = Pediatric Cerebral Performance Category; FSS = Functional Status Scale

**Supplemental Table 6.** Association of Early Epinephrine Bolus with Outcomes in Patients with Hypotension as Immediate Cause of Arrest

| **Outcome** | **Difference (95% CI)** | **Relative Risk (95% CI)** | **P-value** |
| --- | --- | --- | --- |
| **Survival to hospital discharge with favorable neurologic outcome^a^** |  | **1.03 (0.67, 1.58)** | **0.91** |
| Sustained ROSC |  | 1.26 (0.87, 1.83) | 0.22 |
| Survival to hospital discharge |  | 1.07 (0.72, 1.59) | 0.74 |
| Survival to hospital discharge with a PCPC of 1, 2, or no change from baseline |  | 1.06 (0.65, 1.71) | 0.82 |
| Total FSS at hospital discharge | -1.00 (-3.64, 1.63) |  | 0.45 |
| PCPC at hospital discharge | -0.24 (-1.03, 0.56) |  | 0.56 |
| Change from baseline to hospital discharge in FSS of survivors | -0.17 (-2.20, 1.86) |  | 0.87 |
| New morbidity (survivors only) |  | 0.90 (0.47, 1.70) | 0.75 |

Subgroup analysis of the association of early epinephrine bolus (≤2 minutes) with outcomes, controlling for pre-event characteristics. All models control for illness category, Pediatric Risk of Mortality (PRISM) score, vasoactive-inotropic score 2 hours prior to resuscitation, epinephrine infusion at the start of resuscitation.

^a^Favorable neurologic outcome was defined as PCPC of 1, 2, 3, or no change from baseline.

PCPC = Pediatric Cerebral Performance Category; FSS = Functional Status Scale

**Supplemental Table 7.** Association of Early Epinephrine Bolus with Outcomes in Patients with Respiratory Decompensation as Immediate Cause of Arrest

| **Outcome** | **Difference (95% CI)** | **Relative Risk (95% CI)** | **P-value** |
| --- | --- | --- | --- |
| **Survival to hospital discharge with favorable neurologic outcome^a^** |  | **0.85 (0.68, 1.07)** | **0.18** |
| Sustained ROSC |  | 0.87 (0.77, 0.99) | 0.03 |
| Survival to hospital discharge |  | 0.84 (0.69, 1.04) | 0.11 |
| Survival to hospital discharge with PCPC of 1, 2, or no change from baseline |  | 0.94 (0.72, 1.23) | 0.67 |
| Total FSS at hospital discharge | -0.26 (-1.90, 1.37) |  | 0.75 |
| PCPC at hospital discharge | 0.38 (-0.26, 1.01) |  | 0.24 |
| Change from baseline to hospital discharge in FSS of survivors | -0.34 (-1.45, 0.77) |  | 0.55 |
| New morbidity (survivors only) |  | 0.98 (0.53, 1.83) | 0.95 |

Subgroup analysis of the association of early epinephrine bolus (≤2 minutes) with outcomes, controlling for pre-event characteristics. All models control for illness category, Pediatric Risk of Mortality (PRISM) score, vasoactive-inotropic score 2 hours prior to resuscitation, epinephrine infusion at the start of resuscitation.

^a^Favorable neurologic outcome was defined as PCPC of 1, 2, 3, or no change from baseline.

PCPC = Pediatric Cerebral Performance Category; FSS = Functional Status Scale

**Supplemental Table 8.** Association of Early Epinephrine Bolus with Outcomes in Patients with Cardiac Illness Category

| **Outcome** | **Difference (95% CI)** | **Relative Risk (95% CI)** | **P-value** |
| --- | --- | --- | --- |
| **Survival to hospital discharge with favorable neurologic outcome^a^** |  | **1.09 (0.85, 1.39)** | **0.52** |
| Sustained ROSC |  | 1.05 (0.86, 1.28) | 0.65 |
| Survival to hospital discharge |  | 1.03 (0.82, 1.28) | 0.82 |
| Survival to hospital discharge with a PCPC of 1, 2, or no change from baseline |  | 1.32 (0.98, 1.79) | 0.07 |
| Total FSS at hospital discharge | -1.31 (-2.66, 0.05) |  | 0.06 |
| PCPC at hospital discharge | -0.22 (-0.78, 0.35) |  | 0.45 |
| Change from baseline to hospital discharge in FSS of survivors | -0.83 (-2.06, 0.41) |  | 0.19 |
| New morbidity (survivors only) |  | 0.63 (0.42, 0.93) | 0.02 |

Subgroup analysis of the association of early epinephrine bolus (≤2 minutes) with outcomes, controlling for pre-event characteristics. All models control for illness category, Pediatric Risk of Mortality (PRISM) score, vasoactive-inotropic score 2 hours prior to resuscitation, epinephrine infusion at the start of resuscitation.

^a^Favorable neurologic outcome was defined as PCPC of 1, 2, 3, or no change from baseline.

PCPC = Pediatric Cerebral Performance Category; FSS = Functional Status Scale

**Supplemental Table 9.** Association of Early Epinephrine Bolus with Outcomes in Neonates

| **Outcome** | **Difference (95% CI)** | **Relative Risk (95% CI)** | **P-value** |
| --- | --- | --- | --- |
| **Survival to hospital discharge with favorable neurologic outcome^a^** |  | **1.03 (0.68, 1.56)** | **0.88** |
| Sustained ROSC |  | 1.04 (0.71, 1.51) | 0.84 |
| Survival to hospital discharge |  | 1.03 (0.68, 1.56) | 0.88 |
| Survival to hospital discharge with a PCPC of 1, 2, or no change from baseline |  | 1.09 (0.67, 1.78) | 0.72 |
| Total FSS at hospital discharge | -0.68 (-2.63, 0.99) |  | 0.41 |
| PCPC at hospital discharge | -0.07 (-1.27, 1.13) |  | 0.91 |
| Change from baseline to hospital discharge in FSS of survivors | -0.29 (-1.95, 1.37) |  | 0.91 |

Subgroup analysis of the association of early epinephrine bolus (≤2 minutes) with outcomes, controlling for pre-event characteristics. All models control for illness category, Pediatric Risk of Mortality (PRISM) score, vasoactive-inotropic score 2 hours prior to resuscitation, epinephrine infusion at the start of resuscitation.

^a^Favorable neurologic outcome was defined as PCPC of 1, 2, 3, or no change from baseline.

PCPC = Pediatric Cerebral Performance Category; FSS = Functional Status Scale

**Supplemental Table 10.** Cumulative Percent of Subjects with Status Prior to the Current Minute

|  | **Minute** | | | | |
| --- | --- | --- | --- | --- | --- |
|  | **1** | **2** | **3** | **5** | **10** |
| Pulseless | 57 (31%) | 86 (46%) | 100 (54%) | 113 (61%) | 118 (63%) |
| Pulselessness followed by return to bradycardia with poor perfusion | 5 (3%) | 12 (7%) | 23 (12%) | 36 (19%) | 53 (29%) |

Analysis completed among the 186 patients in the cohort with evaluable arterial line waveform data.

**Supplemental Figure 1.** Temporal Evolution of CPR Rhythms and Outcomes, patients receiving early epinephrine

146 events had both invasive BP monitoring and received early epinephrine and were able to be included.

**Supplemental Figure 2.** Temporal Evolution of CPR Rhythms and Outcomes, patients not receiving early epinephrine

40 events had both invasive BP monitoring and received early epinephrine and were able to be included.

**Supplemental Table 11.** Association of Subsequent Pulselessness Status with Outcomes, patients categorized by early epinephrine status

| **Received early epinephrine bolus** | | | | |
| --- | --- | --- | --- | --- |
|  | **Never developed**  **pulselessness**  (n = 52) | **Developed subsequent pulselessness**  (n = 52) | **Developed pulselessness and subsequently had at least one return to bradycardia with poor perfusion**  (n = 42) | **P-value** |
| **Sustained ROSC** | **44 (85%)** | **23 (44%)** | **25 (60%)** | **<0.001** |
| Survival to hospital discharge | 35 (67%) | 25 (48%) | 23 (55%) | 0.131 |
| Survival to hospital discharge with favorable neurologic outcome^a^ | 35 (67%) | 24 (46%) | 22 (52%) | 0.084 |
| Survival to hospital discharge with PCPC of 1, 2, or no change from baseline | 33 (64%) | 21 (40%) | 21 (50%) | 0.063 |
| **Did not receive early epinephrine bolus** | | | | |
|  | **Never developed**  **pulselessness**  (n = 16) | **Developed subsequent pulselessness**  (n = 13) | **Developed pulselessness and subsequently had at least one return to bradycardia with poor perfusion**  (n = 11) |  |
| **Sustained ROSC** | **13 (81%)** | **5 (39%)** | **8 (73%)** | **0.054** |
| Survival to hospital discharge | 10 (63%) | 6 (46%) | 7 (64%) | 0.664 |
| Survival to hospital discharge with favorable neurologic outcome^a^ | 10 (63%) | 6 (46%) | 7 (64%) | 0.664 |
| Survival to hospital discharge with PCPC of 1, 2, or no change from baseline | 9 (56%) | 5 (39%) | 6 (55%) | 0.720 |

ROSC = return of spontaneous circulation; PCPC = Pediatric Cerebral Performance Category

^a^Favorable neurologic outcome was defined as a PCPC of 1, 2, 3, or no change from baseline.
